# Cyanobacterial Neurotoxin BMAA and Mercury in Sharks

**DOI:** 10.3390/toxins8080238

**Published:** 2016-08-16

**Authors:** Neil Hammerschlag, David A. Davis, Kiyo Mondo, Matthew S. Seely, Susan J. Murch, William Broc Glover, Timothy Divoll, David C. Evers, Deborah C. Mash

**Affiliations:** 1 Rosensteil School of Marine and Atmospheric Science, University of Miami, Miami, FL 33149, USA; nhammerschlag@rsmas.miami.edu; 2 Leonard and Jayne Abess Center for Ecosystem Science and Policy, University of Miami, Coral Gables, FL 33124, USA; 3 Department of Neurology, Miller School of Medicine, University of Miami, Miami, FL 33136, USA; d.davis12@med.miami.edu (D.A.D.); kmondo@hiroshima-u.ac.jp (K.M.); mxs1904@miami.edu (M.S.S.); 4 Department of Chemistry, 3247 University Way, University of British Columbia, Kelowna, BC V1V 1V7, Canada; susan.murch@ubc.ca (S.J.M.); williambrocglover@gmail.com (W.B.G.); 5 Biodiversity Research Institute, 276 Canco Road, Portland, ME 04103, USA; tim.divoll@briloon.org (T.D.); david.evers@briloon.org (D.C.E.); 6 Department of Molecular and Cellular Pharmacology, Miller School of Medicine, University of Miami, Miami, FL 33136, USA

**Keywords:** β-*N*-methylamino-l-alanine, conservation, cyanobacteria, total mercury, methylmercury, neurodegenerative disease, neurotoxin, sharks

## Abstract

Sharks have greater risk for bioaccumulation of marine toxins and mercury (Hg), because they are long-lived predators. Shark fins and cartilage also contain β-*N*-methylamino-l-alanine (BMAA), a ubiquitous cyanobacterial toxin linked to neurodegenerative diseases. Today, a significant number of shark species have found their way onto the International Union for Conservation of Nature (IUCN) Red List of Threatened Species. Many species of large sharks are threatened with extinction due in part to the growing high demand for shark fin soup and, to a lesser extent, for shark meat and cartilage products. Recent studies suggest that the consumption of shark parts may be a route to human exposure of marine toxins. Here, we investigated BMAA and Hg concentrations in fins and muscles sampled in ten species of sharks from the South Atlantic and Pacific Oceans. BMAA was detected in all shark species with only seven of the 55 samples analyzed testing below the limit of detection of the assay. Hg concentrations measured in fins and muscle samples from the 10 species ranged from 0.05 to 13.23 ng/mg. These analytical test results suggest restricting human consumption of shark meat and fins due to the high frequency and co-occurrence of two synergistic environmental neurotoxic compounds.

## 1. Introduction

Sharks are exploited in both target fisheries [1,2,3] and as bycatch (both discarded and incidental catch) that is also sold [4,5,6]. The estimates suggest total annual mortality of 100 million sharks killed in 2000 and about 97 million sharks in 2010, with a total range between 63 and 273 million per year [7]. At least 126 countries worldwide catch sharks, and the global annual value of trade in shark parts is approximately $1 billion US. [8]. Though sharks are harvested for meat consumption and/or for their cartilage used in alternative medicine products, the largest driver of shark mortality is directed fishing to obtain their fins for human consumption in shark fin soup [9,10,11,12]. Given their relatively low natural population growth rates, many sharks are undergoing population declines [5,7] rendering about 16% of the ocean’s shark species threatened with extinction [13].

Shark fin soup primarily consumed in China is also a delicacy in other Asian countries and their diaspora communities worldwide [11,12]. Records from the Chinese Song Dynasty (960–1279) describe the use of shark fin soup as a traditional banquet staple [14]. Today, shark fin soup is in increasingly high demand, popular at weddings and other celebrations across Asia [15]. Dietary supplements containing shark cartilage, the health benefits of which are purportedly bolstered by traditional Chinese medicine claims, have gained popularity in western nations. However, the U.S. Food and Drug Administration (FDA) has been unable to confirm any proclaimed benefits [16] and available reports of health benefits are questionable [17]. 

There is growing concern as to the potential negative health consequences associated with consuming shark parts, including fins, meat and cartilage. The neurotoxic compound methyl Hg (MeHg) has been known to bioaccumulate in sharks over their lifespans [18,19,20,21]. As such, Hg levels in shark muscle often exceed advisory guidelines for safe human consumption [21,22,23,24]. For example, the Florida Department of Health (FDOH) advises that people should not eat sharks greater than ~109 cm and further recommends that children and pregnant woman not eat any shark meat [23]. Moreover, recent studies have reported that commercial shark cartilage supplements contain pro-inflammatory compounds that could pose health risks for consumers, especially those with inflammatory diseases [17].

Recently, the cyanobacterial neurotoxin β-*N*-methylamino-l-alanine (BMAA), has been detected in shark fins [25] and shark cartilage supplements [16]. BMAA has been linked to amyotrophic lateral sclerosis/Parkinsonism dementia complex (ALS/PDC) of Guam and has been detected in the brains of North American Alzheimer’s disease and ALS patients [26,27], suggesting that BMAA plays a role as an environmental toxin in neurodegenerative disease. Recent evidence suggests that merely living near a body of water with cyanobacterial blooms, which contaminate the water, fish, and even the air, may increase the risk of developing ALS [28]. In vitro exposures have demonstrated BMAA’s acute neurotoxicity and animal studies show that BMAA exposure leads to motor impairments in rats [29,30]. Thus, the consumption of shark fins and dietary cartilage supplements may pose a risk for human exposure to environmental neurotoxins BMAA and Hg [16,25].

A causal role for BMAA toxicity in humans is still uncertain due to a lack of epidemiological data with human intake estimated from dietary exposures. Thus, it remains unclear whether detection of BMAA in shark fin or cartilage supplements on its own poses a threat to human health. Likewise, it has been noted that the concentrations of MeHg found in fish and marine products are unlikely to cause significant adverse CNS health effects [31]. However, a synergistic toxicity between these two neurotoxic compounds has been suggested, since BMAA concentrations in a range of 10–100 μM were potentiated by MeHg (3 μM) when these were combined. BMAA and MeHg have been shown to decrease the main cellular antioxidant glutationine, which would increase vulnerability of the brain to oxidative stress [31]. Recent studies by Cox and coworkers demonstrate that vervet monkeys fed BMAA for 140 days develop neurofibrillary tangles and β-amyloid deposits in the brain similar to what is seen in patients with neurodegenerative diseases, including ALS and Alzheimer’s disease [32].

Given the potential synergistic toxicity of Hg and BMAA and their likely prevalence in top marine predators, we conducted an expanded analysis to test fin and muscle from an opportunistic sample of 10 different shark species collected from different ocean basins. Shark samples were analyzed for BMAA using high performance liquid chromatography with fluorescence detection (HPLC-FD) and Hg concentrations were quantified by cold vapor atomic fluorescence spectrometry (CVAFS). Since the total Hg (THg) that is measured in shark muscle and fin is mostly in the form of MeHg+, measures of total Hg are generally equivalent to MeHg+ [33,34,35] Here, THg concentrations in fin and muscle samples were measured and compared to MeHg+ for confirmation of levels in select samples. Our results demonstrated that all 10 shark species tested positive for both BMAA and Hg. Independent laboratory confirmation of BMAA and its isomers 2, 4-diaminobutyric acid (DAB) and *N*-(2-aminoethyl) glycine (AEG) was determined by ultra-performance liquid chromatography/mass spectrometry/mass spectrometry (UPLC-MS/MS).

## 2. Results and Discussion

A total of 55 sharks were analyzed for contaminations of BMAA and Hg in selected fin and/or muscle. Our cohort contained 10 different shark species sampled from the Atlantic and the Pacific Ocean (Table 1). These shark species sampled range in threat status from Least Concern (bonnethead shark) to Endangered (great hammerhead) by the International Union for Conservation of Nature (IUCN). Several species (tiger, great hammerhead, and bull) are known to be common in the shark fin trade [12], and the fins and meat of all species sampled are subject to exploitation (Table 1) [36].

**Figure 1:**
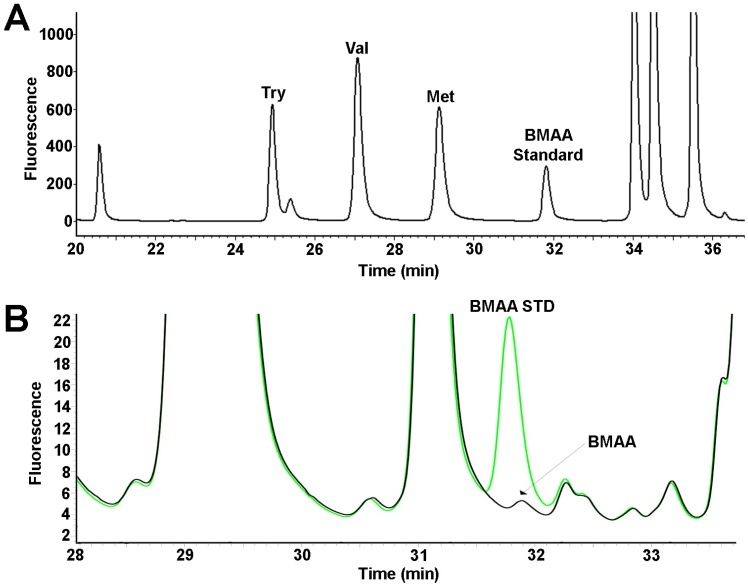
High performance liquid chromatography with fluorescence detection (HPLC-FD) identification of β-*N*-methylamino-l-alanine (BMAA) in shark fins. (**A**) Separation of 6-aminoquinolyl-*N*-hydroxysuccinimidyl carbamate (AQC) derivatized amino acid standards tyrosine (Try), valine (Val), methionine (Met), and BMAA standard; (**B**) representative chromatogram of Australian Tiger shark fin (**black**) and BMAA standard (**green**). Chromatogram shows BMAA has a distinct peak with a retention time of 31.8 mins.

**Figure 2:**
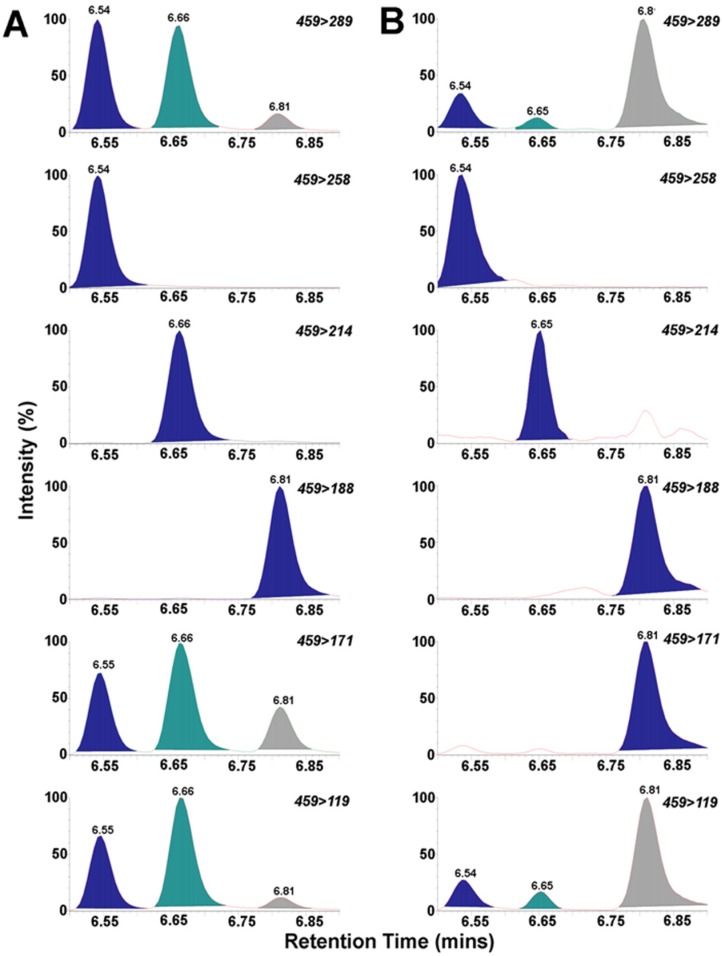
Ultra-performance liquid chromatography/mass spectrometry/mass spectrometry (UPLC-MS/MS) detection and conformation of BMAA in shark fins. (**A**) Chromatograms depicting detection of ACQ derivatized standards of BMAA, and structural isomers *N*-(2-aminoethyl) glycine (AEG) and 4-diaminobutyric acid (DAB); (**B**) UPLC-MS/MS chromatograms of BMAA detection in fins from Australian sharks. The diagnostic selected reaction monitoring (SRM) transitions of the parent ion *m*/*z* 459 to daughter ions 289, 171 and 119 are common to all three isomers. The BMAA (**blue**) peak is selectively identified at 6.55 min by the transition 459 > 258. AEG (**green**) is selectively identified at 6.66 min by the transition 459 > 214. DAB (**grey**) is selectively identified at 6.81 min by the transition 459 > 188.

We used a rapid and sensitive HPLC-FD method for detection of 6-aminoquinolyl-*N*-hydroxysuccinimidyl carbamate (AQC) tagged BMAA (Figure 1) [25]. We detected BMAA in shark fins of all 10 species surveyed in this study in concentrations ranging from 34 to 2011 ng/mg (wet weight) (Table 2). The average concentration for this survey of BMAA in sharks was 366 ± 72 ng/mg (wet weight) (Table 2). The HPLC-FD method has lower sensitivity compared to LC-MS/MS methods [37,38]. The unambiguous detection and identification of BMAA in complex biological samples requires mass spectrometry validation and AQC derivatization to distinguish BMAA from its positional isomers DAB and AEG (Table 3) [16,25]. UPLC-MS/MS was used to confirm the identity of BMAA in a representative sample of shark fins (Figure 2).

**Figure 3:**
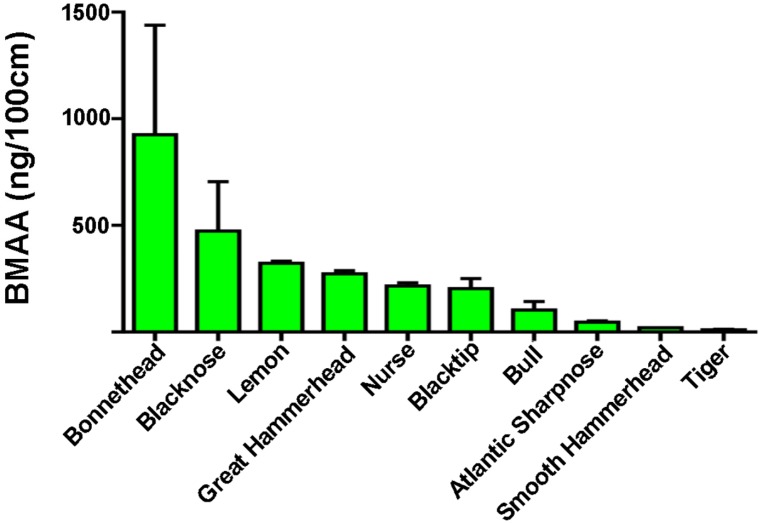
BMAA concentration per unit length of shark fin. Bar graphs depict the mean and standard error of BMAA concentration per 100 cm in ten shark species from the Atlantic and Pacific oceans.

BMAA was below the level of quantitation in only 12% of shark fins tested (Table S1). The highest BMAA concentrations were measured in bonnethead sharks (Figure 3, Table 2; 707 ± 395 ng/mg wet weight and 925 ng/100 cm fin length). This result is in keeping with the elevated levels of BMAA in benthic organisms [39]. The preferred prey of bonnetheads is found in coastal inshore areas that feed primarily on blue cabs (Callinectes sapidus) and other crustaceans [40]. In an examination of cyanobacteria in South Florida and BMAA concentrations in resident fish and invertebrates [41], we found that blue crab and shrimp had among the highest concentrations of BMAA reported in animals (6976 μg/g). Macroalgal abundance per square meter in such habitats can be typically around 20 times higher on the sediment than in the water column [39]. The elevated levels of BMAA may be due to the high occurrence of benthic cyanobacteria associated with the microalgae and detritus that the blue crab and shrimp feed on [41].

Cold vapor atomic fluorescence spectroscopy (CVAFS) and thermal decomposition methods for total Hg in sharks gave positive results in all 10 species surveyed. The THg concentrations ranged from 0.048 to 13.23 ng/mg with a mean concentration of 2.3 ± 0.4 ng/mg (Table 4; Table S2). These values are higher than those reported safe for human consumption, which range from 0.3 to 1.0 µg/g wet weight based on different criteria and benchmark dose estimates reported by health organization or government agencies [42,43]. The highest THg concentrations were found in the bull sharks, averaging 7.26 ± 3.04 ng/mg. Bull sharks are large coastal apex predators with high Hg levels reported previously in agreement with our results [21,44,45]. Shark muscle samples contained nine times the amount of THg as compared to fins (Student’s *t*-test *p* < 0.0001; *n* = 26/20) (Table S1). THg in muscles ranged from 0.27 to 13.23 ng/mg with a mean concentration of 3.8 ± 0.6 ng/mg. In addition, MeHg was measured in a subset of sharks with concentrations ranging from 0.05 to 1.95 ng/mg and a mean concentration of 0.42 ± 0.11 ng/mg (Table 4). THg and MeHg concentration in shark samples tested were highly correlated (Spearman correlations *r* = 0.94, *p* < 0.0001; *n* = 18) in our shark cohort as expected [33,34,35].

Previous studies of MeHg in shark fins and soup reported variable levels, with higher concentrations measured in higher trophic levels species consistent with biomagnification [35]. However, a recent report suggests that THg levels are low in shark fin soup posing only a minor risk for human exposure [35]. We reported that THg and BMAA are detected in shark cartilage dietary supplements [16]. Despite the low levels of THg in shark fin soup, the co-occurrence of BMAA and elemental and Hg in shark fin and muscle should be considered a potential human health concern due to their possible synergistic toxicity to neural tissues [31].

In all shark species surveyed, BMAA levels averaged 15 to 1500 times higher than the concentration of Hg (Table 4). We correlated BMAA and Hg levels by comparing the linear mass density of each contaminant. The concentrations of Hg were positively correlated with BMAA among individuals when both values were normalized for length (concentration/100 cm; Spearman correlations *r* = 0.37, *p* = 0.02; *n* = 39). The positive correlation demonstrates that sharks with higher BMAA concentrations show increased Hg levels. Shark fins are often dried or cooked prior to human consumption. While these preparation methods are known to remove other marine toxins, neither BMAA nor Hg would likely be significantly affected because both are associated with stable incorporation into proteins [46]. BMAA is misincorporated into neural proteins [47,48,49] and Hg-binding proteins are a likely source of harmful accumulation of Hg in the marine food web [50]. 

The sharks surveyed show both inter- and intra-specific variation in BMAA and Hg concentrations. There are various biological and physical properties of the environment that may affect exposures of sharks to spatial and temporal differences in the accumulation of Hg and BMAA through the food web. In the case of Hg, exposure can be through both point source pollution and atmospheric emissions from fossil fuel combustion [51]. The bioaccumulation will depend in part on the presence of anaerobic bacteria that can convert inorganic Hg to the organic form for trophic transfer up the food web to sharks [52] Similarly, variation in the bioaccumulation and exposure of sharks to BMAA will depend on environmental levels of cyanobacteria that increase with nutrient pollution derived from land-based sources [25,41]. While evaluating patterns of spatial, temporal and even individual variation in BMAA and Hg toxicity are important for mitigation efforts to reduce exposure, our data suggest that the risk of BMAA exposure may be greatest between spring and summer seasons (Table S2). Thus, further studies are warranted based on the limited reports of BMAA in the marine food web across diverse geographical locales. 

The prevalence of dementia and Alzheimer’s disease is significantly higher in certain Asian countries [53]. In China alone, the number of people with dementia has increased significantly from 3.7 million in 1990 to 9.2 million in 2010 [54]. Moreover, a report in Lancet on global disease burden found that the number of deaths in China due to Alzheimer’s disease and other dementias doubled between 1990 and 2010, while mortality rates, especially among women, fell steeply during the same period [54]. With the continuing growth of China’s aging population, these findings suggest that the nation is heading for a bigger dementia burden than anticipated [55]. The present study suggests that ingestion of shark fin and shark dietary supplements is a route for human exposure to the environmental toxins BMAA and Hg. Although there are no estimates to help benchmark exposure risk of BMAA to humans from dietary exposures, in China, male infertility has been linked to Hg exposure through consumption of seafood, including shark fins [56]. Because sharks were sampled in South Florida waters, there is a concern of BMAA exposures also to USA residents. In Florida, there is an estimated 0.5 million people over the age of 65 with Alzheimer’s disease. These numbers are anticipated to increase to 0.7 million by the 2020 [57]. 

Given the decline in many shark populations from overfishing, more research is needed to fully understand the potential toxicity of BMAA and Hg to the health and fitness of shark species [1]. Systemic exposure to BMAA and Hg is likely to worsen the problem and limit recovery efforts if not considered in conservation management efforts. Since sharks often occupy high trophic levels in the marine food web, they are vulnerable to bioaccumulation and biomagnification of neurotoxins and other toxic compounds. Given that humans and sharks are both top predators, the results reported here support the view that sharks serve as bioindicators of ecosystem health from human stressors and marine contaminants [58,59].

## 3. Materials and Methods

### 3.1. Sample Collection

Fin clips and muscle biopsies were collected from shark species (*n* = 10) sampled from areas with or without documented cyanobacterial blooms in the Atlantic and Pacific Oceans as described previously [25,26]. Small clips were sampled from archived frozen dorsal fins for analysis of BMAA (*n* = 55), total Hg (*n* = 46). Shark specimens where available were assayed for Hg concentrations in muscle (*n* = 26) and fins (*n* = 20). Tissue specimens from blacknose (*Carcharhinus acronotus*), blacktip (*Carcharhinus limbatus*), bonnethead (*Sphyrna tiburo*), bull (*Carcharhinus leucas*), great hammerhead (*Sphyrna mokarran*), lemon (*Negaprion brevirostris*), nurse (*Ginglymostoma cirratum*), Atlantic sharpnose (*Rhizoprionodon terraenovae*), smooth hammerhead (*Sphyrna zygaena*) and tiger (*Galeocredo cuvier*) sharks were included in this survey (Table 1). 

### 3.2. HPLC Sample Preparation

BMAA in shark fin clips was detected and quantified using high performance liquid chromatography (HPLC) methods as reported previously [25]. Briefly, fin clips (50 mg) were hydrolyzed at 110 °C for 18 h in 6 N HCl (1:8 *w/v*) followed by filtration using centrifugation at 15,800× *g* for 3 min. Sample extracts were concentrated and dried in a speed-vac (Thermo-Savant SC250DDA Speed Vac Plus with a Savant refrigerator trap RVT 4104, ThermoFischer; Waltham, MA, USA). Extracts were re-suspended in 0.1 M trichloroacetic acid and washed with chloroform to remove any residual lipids. The dried extract was resuspended to 1000 µL in 20 mM HCl. A 100 µL aliquot of the sample extract was derivatized with 6-aminoquinolyl-*N*-hydroxysuccinimidyl carbamate (AQC) using the AccQ-Fluor reagent (Waters Crop; Milford, MA, USA). The derivatized samples (20 µL resuspended HCl extract, 60 µL of borate buffer (AccQ-Fluor Reagents A and B; Waters), and 20 µL AccQ-Tag) were run in parallel with buffer and AQC blanks and BMAA, AEG, DAB and reference amino acid standards. The sample matrix was spiked with known amounts of BMAA to determine recovery of the extraction procedure and confirm peak identity. Each sample was prepared in triplicate for quantitative studies and orthogonal detection method comparisons. For the orthogonal method comparisons, the shark samples were prepared by different analysts in different labs as a measure of method ruggedness.

### 3.3. Fluorescence HPLC Methods for Analysis of BMAA

BMAA was separated from amino-acids by reverse-phase high pressure chromatography (Waters Nova-Pak C18 column, 3.9 mm × 300 mm; Waters Crop; Milford, MA, USA) eluted in a gradient of 140 mM sodium acetate, 5.6 mM triethylamine, pH 5.2 (mobile phase A), and 52% (*v/v*) acetonitrile in water (mobile phase B) at 37 °C using a flow rate of 1.0 mL/min, and 10 µL sample injection volume. The samples were eluted using a 60 min gradient: 0.0 min = 100% A; 2 min = 90% A curve 11; 5 min = 86% A curve 11; 10 min = 86% A curve 6; 18 min = 73% A curve 6; 30 min = 57% A curve 10; 35 min = 40% A curve 6; 37.5 min = 100% B curve 6; 47.5 min = 100% B curve 6; 50 min = 100% A curve 6; 60 min = 100% A curve 6. Detection of the AQC fluorescent tag was achieved using a Waters 2475 Multi λ-Fluorescence Detector (Milford, MA, USA) with excitation at 250 nm and emission at 395 nm. Experimental samples were compared with standard spiked shark fin matrix negative for endogenous BMAA and commercial BMAA reference standard (Sigma B-107; >95% purity, St. Louis, MO, USA). The limits of detection (LOD) and limits of quantification (LOQ) were 2.7 and 7.0 ng, respectively. The percentage of recovery of BMAA was 88%.

### 3.4. UPLC/MS/MS of BMAA

BMAA and the isomers *N*-(2-aminoethyl) glycine (AEG) and 2,4-diaminobutyric acid (DAB) were separated, detected and quantified by ultra-performance liquid chromatography/mass spectrometry/mass spectrometry (UPLC/MS/MS) using a fully validated method as previously described [60]. Briefly, 50 mg samples of frozen shark fin clips were accurately weighed and suspended in 1.0 mL of 6 N HCl sealed with N_2_ gas blown into the tubes for 30 s to displace oxygen. Samples were hydrolyzed for 18 h at 110 °C. A subsample of 400 µL was filtered (0.22 μm PVDF Ultrafree MC centrifuge filters; EMD Millipore; Billerica, MA, USA) and a 100 µL aliquot was dried overnight (Labconco Centrivap; Kansas City, MO, USA). The sample was reconstituted in 1.0 mL 20 mM HCl and a 20 µL aliquot was derivatized with 20 µL 6-aminoquinolyl-*N*-hydroxysuccinimidyl carbamate (AQC) in 60 µL borate buffer (AccQ-Fluor Reagents A and B; Waters, Milford, MA, USA). BMAA, AEG and DAB were separated by reverse phase C18 chromatography (BEH column 150 × 2.1 mm 1.7 μm; Waters) and eluted with a gradient of 20 mM ammonium formate with 0.2% formic acid (A) and 0.1% formic acid in acetonitrile; (B). Gradient was delivered by a Waters Acquity I-Class UPLC (Milford, MA, USA) (0 min, 95% A; 1.0 min, 95% A; 7 min, 85% A; 7.5 min, 78% A; 8 min, 15% A; 8.5 min, 15% A; 8.6 min, 95% A; 10 min, 95% A) with a flow rate of 0.7 mL/min at 52 °C. Compounds were clearly separated with BMAA elution at 6.56 min (%RSD = 0.23), AEG at 6.67 min (%RSD = 0.22) and DAB at 6.82 min (%RSD = 0.26) (see Figure 2). Triplicate measures were performed on each shark fin sample (Table 3).

Ions were detected on a triple quadrupole tandem mass spectrometer (Waters Xevo TQS, Milford, MA, USA) with the following parameters: cone voltage was 16 V. Capillary voltage was set to 2500 V with a source offset of 50 V. Desolvation temperature was 550 °C, with a corresponding gas flow of 800 L/h. and a cone gas flow of 150 L/h. Collision-induced-dissociation was performed with 99.999% pure argon pressurized to 7.0 bar with a dwell time of 0.05 s. The characteristic transitions were detected as: BMAA 459 > 258 at collision voltage 18 V, DAB 459 > 188 at collision voltage 20 V, AEG 459 > 214 at collision voltage 20 V. 

### 3.5. Determination of Hg in Fins (CVAFS Method)

Total Hg (THg) includes inorganic and organic forms of Hg. THg and MeHg analyses were performed on shark fin clips following the Standard Operating Procedure modified from the U.S. Environmental Protection Agency (EPA) Test Method 1631 [61]. THg in a sample was isolated and oxidized to mercuric ion using acid digestion, and then reduced to elemental Hg by stannous chloride, purged from the liquid by a carrier gas (Argon). MeHg was extracted from the sample matrix with sodium hydroxide in methanol on a hot block. The Hg species on the traps were desorbed, pyrolyzed and detected by Cold Vapor Atomic Fluorescence Spectrometry (CVAFS) (Millennium Merlin 10.035, PS Analytical, Deerfield Beach, FL, USA). Briefly, the samples (about 0.2 g) were weighed into 10-mL glass ampoules to which 1 mL of deionized water and 2 mL of concentrated HNO_3_ were added. The ampules were then sealed and the samples were autoclaved for 1 h at 105 °C for sample digestion. The samples were diluted with 1% HCl and introduced into CVAFS, reduced with 2% (*v/v*) SnCl_2_ (in 2.5% HCl). Daily analytical runs began with an initial calibration containing 5 non-zero points and a system blank. The mean calibration factor (CFm), calculated from the calibration factor (CFx) for Hg in each of the five standards using the system blank-subtracted peak height, was used for the calculation of sample concentration. Each analytical batch included at least one method blank, a Continuing Calibration Check Samples (CCS), and a Quality Control Sample (QCS). All method blanks during analysis were below the method detection limits (MDLs). The readings of CCS were always within acceptable range (85%–115% for THg of initial calibration). Certified reference material (CRM), DORM-2, was used as a QCS sample throughout the analysis and the recoveries for the QCS samples (84%–128% for THg) were always within acceptable range specified in standard operating procedure (SOPs) (70%–130% for THg). The method limit of detection for the instrument was 0.002 mg/kg.

### 3.6. Determination of Total Mercury in Muscles (Thermal Decomposition Method)

Muscle samples were placed into nickel sample boats, weighed, and analyzed for THg using thermal decomposition technique with an automated direct Hg analyzer (DMA 80, Milestone Incorporated, Shelton, CT, USA) using the US EPA Method 7473 [62]. Assays were run with one sample each of two standard reference materials (DORM-3 and DOLT-4), two method blanks, and one sample blank.

## Figures and Tables

**Table 1:** A summary of shark species, sampling times and locations sites.

Scientific Name	International Union for Conservation of Nature Red List Category	Common Name	Location	Month
*Carharhinus acronotus*	Near Threatened	Blacknose ^b^	25.09417^o^N 81.04234^o^W	March
-	-	Blacknose ^b^	25.00858^o^N 81.00089^o^W	April
-	-	Blacknose ^a^	25.62099^o^N 80.15602^o^W	October
-	-	Blacknose ^a^	Biscayne Bay	June
-	-	Blacknose ^b^	25.09417^o^N 81.04234^o^W	December
-	-	Blacknose ^b^	25.01089^o^N 81.00419^o^W	April
*Carcharhinus limbatus*	Near Threatened	Blacktip ^b^	25.00644^o^N 80.99969^o^W	March
-	-	Blacktip ^b^	25.00644^o^N 80.99969^o^W	September
-	-	Blacktip ^a^	25.59968^o^N 80.15205^o^W	July
-	-	Blacktip ^b^	25.01109^o^N 80.99832^o^W	September
-	-	Blacktip ^b^	25.00644^o^N 80.99969^o^W	March
-	-	Blacktip ^a^	25.62592^o^N 80.15442^o^W	October
-	-	Blacktip ^a^	25.61905^o^N 80.1714^o^W	October
-	-	Blacktip ^a^	25.64757^o^N 80.1881^o^W	April
-	-	Blacktip ^a^	25.67199^o^N 80.18144^o^W	September
-	-	Blacktip ^b^	25.01089^o^N 81.00419^o^W	September
-	-	Blacktip ^b^	25.00976^o^N 81.00079^o^W	September
-	-	Blacktip ^b^	25.00644^o^N 80.99969^o^W	September
-	-	Blacktip ^b^	25.01715^o^N 81.01056^o^W	October
-	-	Blacktip ^b^	25.01715^o^N 81.01056^o^W	February
-	-	Blacktip ^b^	25.01089^o^N 81.00419^o^W	April
-	-	Blacktip ^b^	25.00858^o^N 81.00089^o^W	December
-	-	Blacktip ^b^	25.00623^o^N 80.99723^o^W	March
*Sphyrna tiburo*	Least concerned	Bonnethead ^a^	25.36711^o^N 80.14806^o^W	March
-	-	Bonnethead ^a^	25.36711^o^N 80.14806^o^W	March
-	-	Bonnethead ^a^	25.40807^o^N 80.21806^o^W	October
-	-	Bonnethead ^b^	25.36711^o^N 80.14806^o^W	March
*Carcharhinus leucas*	Near threatened	Bull ^b^	25.01715^o^N 81.01056^o^W	September
-	-	Bull ^b^	25.01309^o^N 80.00129^o^W	September
-	-	Bull ^b^	25.00623^o^N 80.99723^o^W	March
*Sphyrna mokarran*	Endangered	Great Hammerhead ^a^	25.62138^o^N 80.15656^o^W	July
-	-	Great Hammerhead ^b^	25.01715^o^N 81.01056^o^W	September
-	-	Great Hammerhead ^a^	25.740092^o^N 79.967258^o^W	May
-	-	Great Hammerhead ^b^	26.61587^o^N 79.96725^o^W	February
-	-	Great Hammerhead ^b^	26.457892^o^N 80.053938 ^o^W	April
*Negaprion brevirostris*	Near threatened	Lemon ^b^	25.00644^o^N 80.99969^o^W	June
-	-	Lemon ^b^	25.00644^o^N 80.99969^o^W	March
*Ginglymostoma cirraum*	Data deficient	Nurse ^a^	25.61942^o^N 80.1835 ^o^W	September
-	-	Nurse ^b^	24.88335^o^N 80.84475 ^o^W	April
-	-	Nurse ^b^	25.00644^o^N 80.99969 ^o^W	March
-	-	Nurse ^a^	25.62311^o^N 80.15626^o^W	August
-	-	Nurse ^a^	25.60062^o^N 80.15214 ^o^W	August
-	-	Nurse ^a^	25.60569^o^N 80.1534 ^o^W	August
-	-	Nurse ^a^	25.62311^o^N 80.15626 ^o^W	August
-	-	Nurse ^b^	25.00858^o^N 80.00089 ^o^W	September
-	-	Nurse ^b^	Florida Bay	January
-	-	Nurse ^b^	25.00983^o^N 80.99305^o^W	March
*Rhizoprionodon terraenovae*	Least Concerned	Atlantic Sharpnose ^b^	25.00858^o^N 81.00089^o^W	April
-	-	Atlantic Sharpnose ^b^	25.10566^o^N 81.04757^o^W	April
-	-	Atlantic Sharpnose ^b^	Florida Bay	April
*Sphyrna zygaena*	Vulnerable	Smooth Hammerhead ^a^	26.117727^o^N 80.09734^o^W	February
*Galeocerdo cuvier*	Near threatened	Tiger ^c^	−21.12055^o^N 149.22416^o^E	January
-	-	Tiger ^c^	−32.78278^o^N 152.41171^o^E	January
-	-	Tiger ^c^	−24.81665^o^N 152.47257 ^o^E	September
-	-	Tiger ^c^	−24.81665^o^N 152.47257 ^o^E	March

**^a^** Biscayne Bay; ^b^ Florida Bay; **^c^** Pacific Ocean.

**Table 2:** β-*N*-methylamino-l-alanine (BMAA) concentrations detected by high performance liquid chromatography with fluorescence detection (HPLC-FD) in shark fins

Species	Range (ng/mg)	Detected Mean ± SE (ng/mg)	BMAA/Length (ng/100 cm)
Blacknose (*n* = 6) ^a^	ND–1663	573 ± 322 *	473
Blacktip (*n* = 17) ^a^	ND–811	282 ± 72 *	203
Bonnethead (n = 4) ^a^	40–1836	707 ± 395	925
Bull (*n* = 3) ^a^	43–264	180 ± 69	103
Great Hammerhead (*n* = 5) ^a^	42–1528	576 ± 272	273
Lemon (*n* = 2) ^a^	556–628	592 ± 36	322
Nurse (*n* = 10) ^a^	ND–2011	442 ± 315 *	216
Sharpnose (*n* = 3) ^a^	40–115	68 ± 24	47
Smooth Hammerhead (*n* = 1) ^a^	-	43	21
Tiger (*n* = 4) ^b^	34–44	39 ± 2	11

***ND*,** Below limit of detection; **SE:** Standard Error; *: Only detected samples averaged; **^a^:** Atlantic Ocean; **^b^:** Pacific Ocean.

**Table 3:** Comparison of BMAA concentrations detected by HPLC-FD and ultra-performance liquid chromatography/mass spectrometry/mass spectrometry (UPLC-MS/MS).

Species	HPLC-FD * (ng/mg)	UPLC-MS/MS * (ng/mg)
Galeocerdo cuvier	-	-
Tiger ^a^	35.60 ± 1.90	19.20 ± 7.10
Tiger ^a^	31.50 ± 2.60	20.68 ± 3.50
Tiger ^a^	39.60 ± 4.70	33.15 ± 5.60
Tiger ^a^	38.90 ± 5.10	20.17 ± 2.40

* Data presented as Mean ± Standard Error; ^a^: Pacific Ocean; Four replicate biological samples were analyzed in triplicate to determine method reproducibility and ruggedness.

**Table 4:** Mercury concentrations detected in shark fin and muscle.

Species	Range Hg (ng/mg)	THg (ng/mg) *	MeHg (ng/mg) *	BMAA:THg
Blacknose ^a^	0.05–5.65	1.93 ± 2.27 (*n* = 3)	0.71 ± 0.02 (*n* = 2)	429:1
Blacktip ^a^	0.22–7.73	3.70 ± 0.69 (*n* = 16)	1.40 ± 0.75 (*n* = 7)	368:1
Bonnethead ^a^	0.41–1.77	0.96 ± 0.32 (*n* = 4)	0.56 ± 0.44 (*n* = 4)	668:1
Bull ^a^	3.24–13.23	7.26 ± 3.04 (*n* = 3)	2.32 (*n* = 1)	27:1
Great Hammerhead ^a^	-	3.29 (*n* = 1)	N/A	465:1
Lemon ^a^	0.27–1.34	0.81 ± 0.54 (*n* = 2)	0.26 ± 0.08 (*n* = 2)	1390:1
Nurse ^a^	0.06–0.48	0.24 ± 0.04 (*n* = 10)	N/A	1509:1
Sharpnose ^a^	0.44–2.41	1.42 ± 0.98 (*n* = 2)	0.25 (*n* = 1)	70:1
Smooth Hammerhead ^a^	-	2.85 (*n* = 1)	N/A	15:1
Tiger ^b^	0.12–1.61	0.74 ± 0.36 (*n* = 4)	N/A	23:1

* Data presented as mean ± standard error; **^a^:** Atlantic Ocean; **^b^**: Pacific Ocean. N/A, *Samples not available for measurement*.

## Supplementary Materials

The following is available online at www.mdpi.com/2072-6651/8/8/238/s1. Table S1 BMAA and Total Hg concentrations determined by HPLC in sharks. Table S2: BMAA and Mercury concentration detected by seasons in sharks.

## Abbreviations

ALS/PDC	Amyotrophic lateral sclerosis/parkinsonism dementia complex
AEG	*N*-(2-aminoethyl) glycine
AQC	6-aminoquinolyl-*N*-hydroxysuccinimidyl carbamate
BMAA	β-*N*-methylamino-l-alanine
CVAFS	Cold vapor atomic fluorescence spectrometry
CCS	Calibration Check Samples
CFm	Calibration factor
CFx	Calibration factor
DAB	2,4-diaminobutyric acid
FDOH	Florida Department of Health
Hg	Mercury
HPLC-FD	High performance liquid chromatography with fluorescence detection
LOD	Limits of detection
LOQ	Limits of quantification
MDLs	Method detection limits
MeHg	Methyl mercury
QCS	Quality Control Sample
THg	Total mercury
UPLC-MS/MS	Ultra-performance liquid chromatography/mass spectrometry/mass spectrometry
