# Supplementary material for: Cyanobacterial Neurotoxin BMAA and Mercury in Sharks
Source: Toxins (Basel). 2016 Aug 16;8(8):238. doi: 10.3390/toxins8080238 (PMC4999854; doi:10.3390/toxins8080238)
Supplement: Supplementary file 1 [file toxins-08-00238-s001.pdf]

Supplementary Materials: Cyanobacterial Neurotoxin BMAA and Mercury in Sharks

Neil Hammerschlag, David A. Davis, Kiyo Mondo, Matthew S. Seely, Susan J. Murch, William Broc Glover, Timothy Divoll, David C. Evers and Deborah C. Mash

Table S1. β-*N*-methylamino-L-alanine (BMAA) and Total Hg concentrations determined by high performance liquid chromatography (HPLC) in sharks.

Species	Length (cm)	Mean ± SE BMAA (ng/mg)	Total Hg (ng/mg)	BMAA/ Length (ng/100 cm)	Sample Month
Blacknose [b]	122	954 ± 60	*n*/a	782	March
Blacknose [b]	126	161	*n*/a	128	April
Blacknose [a]	120	1663	5.65 [e]	1386	October
Blacknose [a]	120	42.5 ± 1.0	*n*/a	35.4	June
Blacknose [b]	122	43.4 ± 0.9	0.08 [d]	35.6	December
Blacknose [b]	120	ND	0.05 [d]	-	April
Blacktip [b]	61	280 ± 84	0.77 [e]	460	March
Blacktip [b]	69	144 ± 18	1.79 [e]	210	September
Blacktip [a]	162	ND	7.73 [e]	-	July
Blacktip [b]	165	ND	6.55 [e]	-	September
Blacktip [b]	173	286	4.32 [e]	165	March
Blacktip [a]	174	168	3.24 [e]	97	October
Blacktip [a]	177	247	4.35 [e]	140	October
Blacktip [a]	148	794	1.58 [e]	537	April
Blacktip [a]	156	811	0.22 [d]	522	September
Blacktip [b]	165	303	6.89 [e]	184	September
Blacktip [b]	165	745	6.75 [e]	453	September
Blacktip [b]	168	252	4.97 [e]	150	September
Blacktip [b]	166	34.5 ± 0.5	0.22 [d]	20.8	October
Blacktip [b]	122	34.3 ± 0.1	1.95 [d]	28.1	February
Blacktip [b]	188	40.9 ± 0.2	7.43 [e]	21.8	April
Blacktip [b]	152	43.7 ± 0.7	0.46 [d]	28.8	December
Blacktip [b]	148	43.4 ± 0.8	*n*/a	29.3	March
Bonnethead [a]	73.5	632 ± 96	1.77 [e]	860	March
Bonnethead [a]	78.5	320 ± 56	0.50 [e]	408	March
Bonnethead [a]	77	1836 ± 364	1.16 [e]	2385	October
Bonnethead [b]	85	40.3 ± 1.1	0.42 [e]	47.4	March
Bull [b]	163	232 ± 60	13.23 [e]	142	September
Bull [b]	183	264 ± 96	5.33 [e]	144	September
Bull [b]	178	43.0 ± 1.3	3.24 [e]	24.2	March
Great Hammerhead [a]	247	1528 ± 212	3.29 [e]	619	July
Great Hammerhead [b]	181	528 ± 210	*n*/a	411	September
Great Hammerhead [a]	175	719 ± 21	*n*/a	291	May
Great Hammerhead [b]	310	63.1 ± 0.9	*n*/a	20.4	February
Great Hammerhead [b]	396	41.5 ± 3.6	*n*/a	23.7	April
Lemon [b]	168	556 ± 210	1.34 [e]	332	June
Lemon [b]	201	628 ± 66	0.27 [e]	312	March
Nurse [a]	226	223	0.18 [d]	99	September
Nurse [b]	213	169	0.10 [d]	79	April
Nurse [b]	168	161	0.36 [d]	96	March

Table S1. *Cont.*

Species	Length (cm)	Mean ± SE BMAA (ng/mg)	Total Hg (ng/mg)	BMAA/ Length (ng/100 cm)	Sample Month
Nurse [a]	165	ND	0.13 [d]	-	August
Nurse [a]	235	ND	0.06 [d]	-	August
Nurse [a]	207	ND	0.22 [d]	-	August
Nurse [a]	241	ND	0.26 [d]	-	August
Nurse [b]	169	2010.5 ± 15.5	0.37 [d]	952	September
Nurse [b]	89	55.2 ± 6.4	0.28 [d]	47.8	January
Nurse [b]	250	47.1 ± 1.2	0.48 [d]	18.8	March
Atlantic Sharpnose [b]	88	40.2 ± 0.5	0.44 [e]	45.7	April
Atlantic Sharpnose [b]	120	47.53	*n*/a	39.6	April
Atlantic Sharpnose [b]	208	115.0 ± 0.1	2.41 [e]	55.3	April
Smooth Hammerhead [a]	207	42.6 ± 0.2	2.85 [e]	20.6	February
Tiger [c]	390	35.6 ± 1.9	1.61 [d]	9.12	January
Tiger [c]	349	31.5 ± 2.6	0.31 [d]	9.02	January
Tiger [c]	330	39.6 ± 4.7	0.92 [d]	12	September
Tiger [c]	253	38.9 ± 5.1	0.12 [d]	15.43	March

SE: Standard Error; **ND:** Below the level of detection; ***n*/a:** *Samples not available for analysis;* **-:** *Undetermined;*
[a] Biscayne Bay; [b] Florida Bay; [c] East Australian Coast; [d] Fin; [e] Muscle

Table S2. BMAA and Mercury concentrations detected by sessions in sharks.

Sample Season	BMAA * (ng/mg)	THg (ng/mg)
Winter [a]	43.7 ± 3.8 (n = 8)	1.1 ± 0.4 (n = 7)
Spring [b]	254.6 ± 62.4 (n = 22)	1.5 ± 0.5 (n = 16)
Summer [c]	708.8 ± 435.6 (n = 3)	1.9 ± 1.1 (n = 7)
Fall [d]	593.8 ± 164.2 (n = 16)	3.9 ± 0.9 (n = 16)

* Only detected samples averaged; [a] December–February; [b] March–May; [c] June–August; [d] September–November
